# Effectiveness and cost-effectiveness of the PLAN-A intervention, a peer led physical activity program for adolescent girls: results of a cluster randomised controlled trial

**DOI:** 10.1186/s12966-021-01133-8

**Published:** 2021-05-13

**Authors:** Russell Jago, Byron Tibbitts, Kathryn Willis, Emily Sanderson, Rebecca Kandiyali, Tom Reid, Ruth R Kipping, Rona Campbell, Stephanie J MacNeill, William Hollingworth, Simon J. Sebire

**Affiliations:** 1 Centre for Exercise, Nutrition & Health Sciences, School for Policy Studies, University of Bristol, 8 Priory Road, BS8 1TZ Bristol, UK; 2 The National Institute for Health Research Applied Research Collaboration West (NIHR ARC West) at University Hospitals Bristol NHS Foundation Trust, Bristol, UK; 3 Bristol Trials Centre, Bristol Randomised Trials Collaboration, University of Bristol, Bristol, UK; 4 Bristol Medical School: Population Health Sciences, University of Bristol, Bristol, UK

**Keywords:** Physical activity, Peers, Adolescent girls, Intervention, School

## Abstract

**Background:**

Physical activity is associated with improved health. Girls are less active than boys. Pilot work showed that a peer-led physical activity intervention called PLAN-A was a promising method of increasing physical activity in secondary school age girls. This study examined the effectiveness and cost-effectiveness of the PLAN-A intervention.

**Methods:**

We conducted a cluster randomised controlled trial with Year 9 (13–14 year old) girls recruited from 20 secondary schools. Schools were randomly assigned to the PLAN-A intervention or a non-intervention control group after baseline data collection. Girls nominated students to be peer leaders. The top 18 % of girls nominated by their peers in intervention schools received three days of training designed to prepare them to support physical activity. Data were collected at two time points, baseline (T0) and 5–6 months post-intervention (T1). Participants wore an accelerometer for seven days to assess the primary outcome of mean weekday minutes of moderate-to-vigorous physical activity (MVPA). Multivariable mixed effects linear regression was used to estimate differences in the primary outcome between the two arms on an Intention-to-Treat (ITT) basis. Resource use and quality of life were measured and a within trial economic evaluation from a public sector perspective was conducted.

**Results:**

A total of 1558 girls were recruited to the study. At T0, girls in both arms engaged in an average of 51 min of MVPA per weekday. The adjusted mean difference in weekday MVPA at T1 was − 2.84 min per day (95 % CI = -5.94 to 0.25) indicating a slightly larger decline in weekday MVPA in the intervention group. Results were broadly consistent when repeated using a multiple imputation approach and for pre-specified secondary outcomes and sub-groups. The mean cost of the PLAN-A intervention was £2817 per school, equivalent to £31 per girl. Economic analyses indicated that PLAN-A did not lead to demonstrable cost-effectiveness in terms of cost per unit change in QALY.

**Conclusions:**

This study has shown that the PLAN-A intervention did not result in higher levels of weekday MVPA or associated secondary outcomes among Year 9 girls. The PLAN-A intervention should not be disseminated as a public health strategy.

**Trial registration:**

ISRCTN14539759–31 May, 2018.

**Supplementary Information:**

The online version contains supplementary material available at 10.1186/s12966-021-01133-8.

## Background

Physical activity is associated with lower levels of risk factors for cardio-metabolic diseases and better mental health among adolescents [1–3]. The amount of physical activity in which young people engage decreases with age and a number of studies have shown that large proportions of adolescents do not meet the current public health guidance of an hour of moderate-to-vigorous intensity physical activity (MVPA) per day [4–6]. This issue is particularly acute among girls, with girls less active than boys from the start of primary school and the slope of decline being steeper than boys throughout adolescence [4–6]. There is a need to find ways to help girls to be more physically active.

Schools are a key site for public health interventions as most children attend full-time education and schools have space, resources and staff to deliver health improvement programmes. The overall impact of school-based physical activity interventions has been very small, as assessed through meta-analysis [7].The majority of these interventions have employed educational components or re-formatting of educational structures such as modifications to physical education lessons rather than changes to school policies or the school environment. The limited effectiveness of these programmes has led to calls for more novel school-based approaches to increase physical activity for girls [8].

A largely under-explored approach to increasing physical activity among girls is via peer supporters, which is a method that has been shown to be effective to reduce smoking in the ASSIST (A Stop Smoking In Schools Trial) programme [9]. ASSIST is based on Diffusion of Innovation Theory (DOI) which suggests that key influencers can relay messages and social norms through a group or organization such as a school peer group. We have adapted the key approaches from the ASSIST programme to develop a new school-based physical activity intervention called PLAN-A (Peer-Led physical Activity iNtervention for Adolescent girls) [10]. PLAN-A brings together DOI [11] with key aspects of Self-Determination Theory (SDT) [12, 13] to provide choice and autonomy over physical activity while also building physical activity competence and connectedness with others. In an earlier feasibility trial of the PLAN-A intervention we showed in 6 schools (4 intervention, 2 control) that there was a 6.1 min (95 % CI = 1.4 to 10.8 min) difference in daily MVPA 12 months after baseline measures which favoured the intervention group [10]. However, it was important to examine whether these effects were maintained when scaled up to a larger number of schools in a wider range of localities. The aim of this cluster-randomised controlled trial was to evaluate whether PLAN-A is effective and cost-effective at increasing adolescent girls’ (13–14 years) physical activity. A mixed-methods process evaluation was conducted concurrently [14], the results of which will be published separately in the study monograph.

## Methods

This study followed the Consolidated Standards of Reporting Trials (CONSORT) extension to cluster randomised trials guidelines [15]. The PLAN-A intervention was evaluated via a two-arm, cluster randomised controlled trial. School was the unit of allocation (cluster) and outcomes were assessed at baseline (T0: Autumn term of Year 9 in 2018) and follow-up (T1: Autumn term of Year 10 in 2019, 5–6 months post-intervention and approximately 12 months post-baseline) [14]. Eligible schools were state-funded secondary schools recruited from three broad regions in the Southwest of England (Avon, Devon and Wiltshire). Allocation in a 1:1 ratio was stratified by region and the proportion of pupils receiving free school meals in each school to ensure balance within each stratum. Computer-based allocation and statistical analyses of outcomes were conducted by a member of the Bristol Randomised Trials Collaboration (BRTC: a UKCRC-registered Clinical Trials Unit) who was blind to school identity and independent of the fieldwork team. Pupils and teachers were, by necessity, not blinded to allocation but were blinded to the identity of other schools in the study. At baseline, participants were Year 9 girls within the schools. All Year 9 girls attending school were eligible to participate. All 20 schools received £500 at the end of data collection as recompense for staff time and pupils received a £10 gift voucher for taking part in each of the two assessments.

### Sample size

A detailed description of the power assumptions and calculations has been reported previously [14]. Briefly, we evaluated several different scenarios based on detecting a difference of between 6 and 10 min in weekday MVPA, a standard deviation in MVPA of 20 based on results from the feasibility study[10], an intra-cluster coefficient of between 0.01 and 0.001 and coefficient of variation in cluster size of 0.22. After accounting for potential variations in school size, up to 30 % of participants not providing outcome data, 90 % power and alpha of 0.05 or 0.01, we estimated that between 10 and 18 schools would be required. We therefore recruited 20 schools to allow for schools to drop out whilst preserving ample statistical power.

### Intervention content

Detailed descriptions of the intervention content have been provided previously [14, 16]. The intervention had four key components.

1) All Year 9 girls took part in a peer nomination process in which they identified influential pupils in the year group. In each school, the 18 % of pupils receiving the most nominations were invited to become a peer supporter in the study (3 pupils across all 10 intervention schools declined the offer to be a peer supporter).

2) PLAN-A trainers (freelance females with backgrounds in physical activity promotion and/or education) received training in intervention delivery during a three-day train-the-trainer program. The training covered the key aspects of SDT theory, practiced delivery of the peer-supporter training and other key issues such as managing challenging behaviour.

3) Trainers delivered a two-day training program to the peer supporters for each intervention school, preferably off-site. The training covered why physical activity is important, the way girls could choose to be active, how to initiate conversations with peers about physical activity and how to encourage peers to be active. A supplementary booklet and diary was given to each peer supporter to use during the training and then to record peer-supporting activity later. There was then a booster day for all intervention schools, half-way through the 10-week intervention period (see below) that reiterated the same content and sought to solve any problems or challenges that the peer supporters may have faced.

4) Peer supporters were encouraged to informally promote physical activity among their peer group for 10-weeks.

### Usual physical education provision

Schools in both the intervention and control group were encouraged to continue with usual physical education provision.

### Data collection

In both intervention and control groups, data were collected at T0 and T1 in schools during school hours by the study fieldwork team. The following descriptive variables were self-reported at T0 only: (1) home postcode to derive Index of Multiple Deprivation (IMD), a national measure of socio-economic position for England based on place, (2) date of birth, (3) ethnicity (White, Mixed, Asian/Asian-British, Black/African/Caribbean/Black British, Other), (4) family affluence (low affluence 0–9 high affluence) [17] and receipt of free school meals a proxy measure of household income. All other measures were collected at both T0 and T1.

The primary outcome was accelerometer-assessed mean weekday minutes of MVPA. Participants wore an ActiGraph wGT3X + for seven consecutive days. Periods of ≥ 60 min of zero counts were classified as ‘non-wear’ and removed from analyses. Participants were included in the primary analysis if they provided ≥ 2 valid weekdays of data (≥ 500 min of data between 06:00 and midnight). Mean minutes of weekday MVPA were calculated using the Evenson [18] threshold of minutes with ≥ 2296 counts per minute. We also calculated mean minutes of weekend MVPA and mean weekday and weekend minutes of sedentary time using the Evenson cut-point of < 100 counts per minute which were secondary outcomes. (Participants were included in the weekend data if they provided day for a Saturday or Sunday with at least 500 min of valid data). Other secondary outcomes included health-related quality of life and self-esteem. Health related quality of life was measured via the EuroQol five-dimensional youth questionnaire (EQ-5D-Y [19]) and KIDSCREEN-10 [20]. Neither of these measures have a validated set of preference weights for use in economic evaluation, therefore we also present mapped Child Health Utility (nine-dimensional) questionnaire (CHU-9D) scores derived from the KIDSCREEN-10 [21]. Self-esteem was assessed using the Self-Description Questionnaire [22].

Several hypothesised potential psychosocial mediators of behaviour change were key to our logic model of how the intervention would work (Web Appendix 1). Motivation types (intrinsic, identified, introjected, external, amotivation) were assessed using the Behavioural Regulation in Exercise Questionnaire-2, BREQ-2 [23]. Psychological needs satisfaction of SDT constructs was measured using validated scales for autonomy [24], competence [25] and relatedness [24]. Eight items were used to assess participants’ self-efficacy to be physically active in different situations [26]. Social support for physical activity was assessed using six items from a broader questionnaire measuring factors associated with physical activity in adolescents[27] at T0 and T1 and peer support for PA was assessed using two questions that were created for PLAN-A: (1) Has anyone in your year group talked with you recently about physical activity? (Yes; No; I’m not sure) and (2) Did talking to anyone in your year help you to be more active? (Yes; No; I’m not sure; I didn’t speak to anyone).

### Statistical Analysis

Descriptive statistics (means, standard deviations, frequencies and percentages) were calculated to describe the sample. The primary analysis was conducted on an Intention-to-Treat (ITT) basis including all participants providing complete data. Only participants providing T0 data were asked to provide data at T1. Multivariable mixed effects linear regression was used to estimate difference in mean weekday minutes of MVPA, between intervention and control groups at T1 adjusting for T0 outcome score, number of valid days of accelerometer data available and stratification variables [28]. To account for clustering by school, this analysis included a random effect for which school the pupil attended. Sensitivity analysis was performed accounting for variables which were imbalanced at baseline. Baseline imbalance is determined using a rule of a difference in a variable between arms greater than half a standard deviation or 10 %. Comparable analyses were conducted for all secondary outcomes [28]. Psychosocial mediator variables were summarised using means and standard deviations at T0 and T1. Mediation analysis was conducted to explore whether any effect of the intervention was mediated by self-determined physical autonomous motivation, autonomy, competence and relatedness. Mediators were treated as continuously measured variables and were described using the mean scores stratified by intervention and control group. First, the correlation between mediators and the outcome measurement was calculated at baseline and at follow-up. Then a mediation analysis was performed using the medeff command in Stata [29] using cluster-robust standard errors and mediators at T1. This analysis adopts a causal mediation approach and allows for the estimation of the total effect (average effect of the intervention on the outcome), average direct effect (average intervention effect working through all mechanisms excluding the mediator), the average causal mediation effect (average intervention effect through the mediator) and the proportion of effect mediated (fraction of the total effect that is explained by the mediator) of individual psychosocial variables on the primary outcome. Models included the baseline values of the mediator and primary outcome, treatment arm, number of weekdays used to derive the primary outcome, area (Avon, Devon or Wiltshire) and school-level index of multiple deprivation used in the randomisation. All results from these models are presented with 95 % confidence intervals. Multiple imputation using chained equations (MICE) was performed and the primary analysis repeated using imputed data. The imputation model used linear regression and included variables used in the randomisation, school, number of days of valid MVPA data at baseline, baseline weekday MVPA, variables found to be imbalanced at baseline and baseline variables which were different between those pupils with missing versus non-missing primary outcome data using a threshold of p < 0.1.

Prespecified subgroup analyses were performed by including an interaction term between the intervention arm and subgroup variable to estimate whether the intervention was differentially effective in subgroups of socioeconomic position using the following variables: pupil-level free school meals (yes/no); school-level proportion of free school meals as a continuous measure; pupil-level IMD quintile (based on home postcode); school-level weighted IMD (based on postcode of the school). Interaction terms were also estimated by subgroups based on median distance (by school) between pupil home postcode and school postcode); nominated peer supporters (peer supporters vs. girls not nominated as peer supporters; all schools had nominated peer supporters, only those in the intervention schools received intervention training) and the proportion of sedentary time at baseline as a continuous measure. We conducted further subgroup analyses post hoc, assessing whether there was a differential effect of the intervention according to whether or not pupils met the UK Chief Medical Officer’s guidelines of an average of 60 min of MVPA per day [30] at baseline and the mode of transport used (active vs. not active) to travel home from school at baseline. In further post hoc analyses, mean weekday and weekend counts per minute (CPM) at T1 were compared between arms.

### Economic analyses

 We conducted a within-trial economic evaluation from the perspective of the public sector funder (school and local authority) using 2019 prices and UK GBP. The primary economic outcome was cost per unit change in weekday MVPA.

Mean and incremental intervention costs, outcomes (KIDSCREEN-10, mapped CHU-9D, EQ-5D visual analogue scale (VAS)) and quality-adjusted-life-years (QALYs) per pupil were estimated from baseline to follow-up. Resource use data including intervention materials, venue costs, staff and trainer time, expenses, travel and administration costs were collected prospectively using data collection forms and expense claims completed by the research team, trainers and school contacts. Unit costs were estimated from published sources including the national teachers’ pay scale (https://www.unison.org.uk). As costs and outcomes were only observed for a 12-month period, discounting was not applied.

Cost-effectiveness was summarised in terms of incremental cost-effectiveness ratios (ICERs). Uncertainty (95 % CI) in ICERs was obtained from bootstrapped samples using a two-stage procedure with shrinkage correction for clustered outcome data [31, 32]. The uncertainty in cost per improvement in MVPA and cost per QALY gained were summarised using cost-effectiveness acceptability curves [33].

## Results

**Fig. 1:**
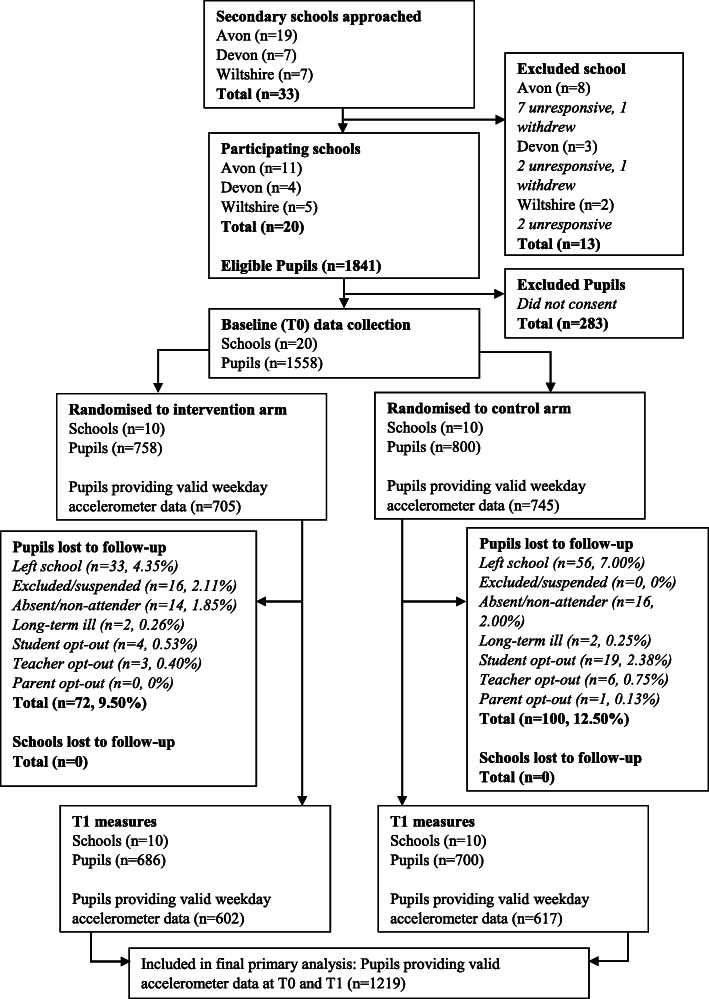
CONSORT Flow Diagram

Twenty schools were recruited with 1558 girls of which 758 (48.6 %) girls were in the 10 schools allocated to the intervention arm (Fig. 1). The school size (Year 9 girls on the roll) ranged from 63 to 152 (mean 95.4, Standard deviation 24.2). 1219 girls (78.2 %) provided valid accelerometer data at T0 and T1. The sample was predominately white (88.5 %) which reflects the ethnic distribution of Southwest England (Table 1). 167 (10.8 %) were receiving free school meals but there was a spread of participants across IMD quintiles. There were, however, some imbalances between study arms at baseline on pupil characteristics. The control arm had fewer girls in the most deprived quintile and more girls in the least deprived quintile than the intervention arm (most deprived: 21.51 % in the intervention arm vs. 8.77 % in the control arm; least deprived: 15.28 % vs. 27.67 %). There were also twice as many girls receiving free school meals in the intervention arm (14.08 % vs. 7.63 %). Finally, a higher proportion of girls in the control arm used active travel as means to get to (58.38 % vs. 50.00 %) and from school (66.01 % vs. 58.33 %).

**Table 1:** Characteristics of sample at T0 (baseline)

Variable	Overall ***N*** =1558	Intervention arm (N schools= 10; N pupils=758)		Control arm (N schools= 10; N pupils=800)	
Age in years; mean (SD)	1311	624	13.80 (0.33)	687	13.80 (0.31)
Area; N(%)	1558	758		800	
Avon			387 (51.06)		381 (47.63)
Devon			207 (27.31)		164 (20.50)
Wiltshire			164 (21.64)		255 (31.88)
Ethnicity; N(%)	1553	753		800	
White			659 (87.52)		716 (89.50)
Mixed			54 (7.17)		54 (6.75)
Asian/Asian-British			19 (2.52)		16 (2.00)
Black/African/Caribbean/Black British			20 (2.66)		13 (1.63)
Other			1 (0.13)		1 (0.13%)
Pupil IMD Quintile n(%)	1415	674		741	
1 (most deprived)			145 (21.51)		65 (8.77)
2			111 (16.47)		99 (13.36)
3			149 (22.11)		172 (23.21)
4			166 (24.63)		200 (26.99)
5 (least deprived)			103 (15.28)		205 (27.67)
Family affluence; mean (SD)	1558	758	6.67 (1.89)	800	7.01 (1.68)
Receiving free school meals	1553	753		800	
No; N(%)			637 (84.59)		734 (91.75)
Yes; N(%)			106 (14.08)		61 (7.63)
Rather not say; N(%)			10 (1.33)		5 (0.63)
Travel mode to school					
Walk; N(%)	1556	756	365 (48.28)	800	459 (57.38)
Cycle; N(%)			13 (1.72)		8 (1.00)
Car; N(%)			227 (30.03)		216 (27.00)
Bus/train; N(%)			151 (19.97)		117 (14.63)
Travel mode from school					
Walk; N(%)	1556	756	428 (56.61)	800	521 (65.13)
Cycle; N(%)			13 (1.72)		7 (0.88)
Car; N(%)			153 (20.24)		137 (17.13)
Bus/train; N(%)			162 (21.43)		135 (16.88)

Average valid accelerometer wear time at T0 and T1 is reported in Table 2. At T0, girls in both arms engaged in an average of 51 min of MVPA per weekday. This reduced to 49 min in the control group and 45 min in the intervention group at T1 (Table 2). The adjusted mean difference in weekday MVPA at T1 was − 2.84 min per day (95 % CI = -5.94 to 0.25). This indicates that while there was a slightly larger decline in weekday MVPA in the intervention group than in the control group, there was not strong evidence that the between-arm difference differed from zero. Similar findings were provided for all secondary outcomes providing an overall indication that intervention did not have a beneficial impact on the participants’ physical activity or self-esteem. Web appendix 2 provides the data for the complete case (*n* = 1062) and multiple imputation results (*n* = 1558). When additionally adjusted for variables which were imbalanced at baseline (free school meals, travel to and from school and IMD quintile) the complete case results provided evidence of a small difference in means in the physical activity levels of the girls (-3.57 min per day, 95 % CI = -6.75 to -0.39), and multiple imputation model revealed a smaller difference still (-2.54 min per day, -5.67 to 0.59). In a post hoc analysis comparing mean weekday CPM at T1 between arms the results mirrored the primary outcome (web appendix 2).

**Table 2:** – Main trial outcomes and secondary outcomes

Variable		Intervention		Control		Difference between intervention and control^c^	
		n	Median (IQR)	n	Median (IQR)		
Average n valid^a^ weekdays	T0	732	4 (4,5)	779	4 (4,5)		
	T1	667	4 (3,5)	689	4 (3,5)		
		n	Mean (SD)	n	Mean (SD)	Difference (95 % CI)	*p*-value
Weekday MVPA minutes^b^	T0	693	51.03 (20.47)	738	51.41 (20.10)	-2.84 (-5.94, 0.25)	0.071
	T1	603	45.19 (18.43)	616	48.89 (20.85)		
Weekend MVPA mins	T0	496	3437 (25.42)	526	35.71 (27.09)	-0.97 (-11.49, 9.55)	0.857
	T1	347	41.50 (55.57)	386	35.66 (31.68)		
Weekday sedentary	T0	693	590.80 (93.74)	738	591.73 (92.82)	2.51 (-12.37, 17.38)	0.741
	T1	603	595.88 (100.28)	616	589.95 (96.70)		
Weekend sedentary	T0	493	527.12 (110.06)	526	521.05 (101.08)	3.44 (-22.03, 28.91)	0.791
	T1	347	528.45 (122.56)	386	535.79 (115.78)		
Self-esteem	T0	729	4.30 (1.10)	773	4.38 (1.11)	0.022 (-0.11, 0.16)	0.741
	T1	669	4.14 (1.19)	677	4.15 (1.11)		

*IQR* Interquartile range

^a^ Valid day criteria: ≥500 min of wear time between 05:00am and 11:59pm

^b^Primary outcome

^c^Analyses adjusted for baseline measure of the outcome and variables used in the randomisation. Measures of MVPA and minutes of sedentary activity additionally adjusted for the number of valid days of data

**Fig. 2:**
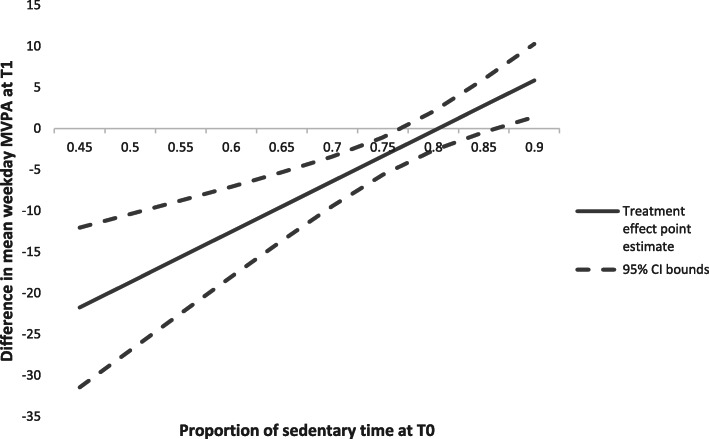
Treatment effect over differing levels of baseline sedentary time. Figure 3 shows the point estimate of the treatment effect (difference in mean weekday MVPA at T1) as a linear function of the proportion of sedentary time reported at T0 along with the 95% confidence interval bounds. We are not powered to draw conclusions from these estimates or confidence intervals, but this analysis could suggest that the Plan-A intervention has a more positive effect on those who have higher proportions of sedentary time prior to the intervention.

There was evidence that PLAN-A differentially affected MVPA in two of the six pre-specified subgroups; nominated peer supporters, and those with high sedentary time at baseline. This evidence was assessed based on the p-value of the likelihood ratio test being less than 0.05. The likelihood ratio test compares a model with, and one without, an interaction term between the subgroup variable and the intervention arm (Table 3). Post hoc data analyses indicated that in the peer supporter subgroup the decline in MVPA was more pronounced than in the non-peer supporter group (difference in treatment effect between nominated peer supporters and non-nominated supporters in all 20 schools was estimated as -4.08, 95 % CI =-8.14 to -0.01), suggesting a small possibility of a negative treatment effect. However, as a post hoc analysis this result should be treated with caution. For pupils who had low (< 75 %) sedentary time at baseline (34.5 % of the sample), the difference in means was negative, meaning that pupils in the intervention arm were estimated to have lower weekday MVPA at T1 than those in the control arm (Fig. 2). For pupils who had higher (≥ 85 %) sedentary time at baseline (4.7 % of the sample) the difference in means was positive, meaning that pupils in the intervention arm were estimated to have higher weekday MVPA at T1 than those in the control arm.

**Table 3:** Effect modification analyses

Baseline Characteristics	LRT *p*-value for interaction term between baseline characteristic and treatment group	Outcome of subgroup analysis
Proportion of free school meals (school level)	0.884	The model with the interaction term does not do a better job of explaining the data than the model without the interaction term
Median distance of home to school (school level)	0.371	
IMD (school level): weighted IMD	0.885	
IMD (pupil level): IMD quintile	0.368	
Meeting CMO guidelines of an average of at least 60 min MVPA per day on weekdays (pupil level)	0.138	
Mode of transport from school (pupil level): active travel (walk or cycle/scoot) vs. non-active travel (car or bus/train)	0.658	
Nominated peer supporter	0.047	The model with the interaction term does a better job of explaining the data than the model without the interaction term. Treatment effect among those who were nominated peer supporter (in intervention and control schools) is -5.98 (-10.34, -1.61) *p* < 0.01. Treatment effect among those who were non-nominated peer supporter (in intervention and control schools) is -1.90 (-5.18, 1.38) *p* = 0.26. Difference in treatment effect between nominated peer supporters and non-nominated supporters is estimated as -4.08 (-8.14, -0.01) *p* = 0.049
Proportion of sedentary time at baseline	< 0.001	The model with the interaction term does a better job of explaining the data than the model without the interaction term. Proportion of sedentary time at baseline is a continuous measure. The difference in treatment effect is a function of proportion of sedentary time at baseline: -49.34 + 61.32*(Proportion of sedentary time at baseline).

*LRT* likelihood-ratio test

All psychosocial mediator variables, except for controlled motivation, showed similar small deteriorations in each arm between T0 and T1 (Table 4). Controlled motivation (i.e. pupils feeling more motivated by guilt or pressure) did not change over time in the intervention group but showed a small increase from T0 to T1 in the control group. No meaningful correlations between the potential mediators and weekday MVPA (primary outcome) were observed, nor evidence of mediation; the confidence interval of the average causal mediated effect for each potential mediator included zero (Web appendix 3). Peer support for PA also deteriorated between T0 and T1 equally between arms. For question (1) ‘yes’ responses stayed similar between time points in both arms (37.75–37.98 % in the intervention arm, 40.78–39.63 % in the control arm) but ‘no’ responses increased (29.01–36.66 % in the intervention arm, 28.61–33.43 % in the control arm). For question (2) ‘yes’ responses decreased in both arms between T0 and T1 (20.24–18.86 % in the intervention arm, 21.93–18.25 % in the control arm), ‘no’ responses increased (22.49–29.68 % in the intervention arm, 23.31–29.89 % in the control arm), and ‘I didn’t speak to anyone’ also increased similarly (23.02–25.44 % in the intervention arm, 23.43–25.72 % in the control arm).

**Table 4:** Psychosocial variable means at T0 and T1 by trial arm

Variable	Intervention		Control	
	N	Mean (SD)	N	Mean (SD)
Physical activity motivation: Autonomous				
T0	731	2.49 (0.99)	779	2.49 (1.01)
T1	677	2.39 (1.02)	687	2.35 (1.01)
Physical activity motivation: Controlled				
T0	739	1.35 (0.81)	786	1.31 (0.82)
T1	681	1.35 (0.85)	692	1.37 (0.84)
Physical activity psychological need satisfaction: Autonomy				
T0	737	5.08 (1.39)	786	5.14 (1.37)
T1	675	4.89 (1.32)	696	4.97 (1.35)
Physical activity psychological need satisfaction: Competence				
T0	743	4.49 (1.58)	786	4.38 (1.58)
T1	678	4.31 (1.49)	690	4.12 (1.49)
Physical activity psychological need satisfaction: Relatedness				
T0	733	4.80 (1.74)	779	4.83 (1.75)
T1	681	4.68 (1.75)	686	4.64 (1.71)
PA self-efficacy				
T0	734	1.38 (0.42)	786	1.40 (0.44)
T1	675	1.29 (0.47)	684	1.31 (0.46)
Physical activity social support				
T0	741	1.46 (0.68)	781	1.41 (0.64)
T1	679	1.33 (0.65)	691	1.25 (0.66)
Peer norms for physical activity: prevalence				
T0	744	1.48 (0.63)	787	1.47 (0.64)
T1	681	1.35 (0.66)	697	1.38 (0.68)
Peer norms for physical activity: importance				
T0	745	1.44 (0.86)	790	1.49 (0.89)
T1	681	1.34 (0.85)	693	1.30 (0.87)
Peer norms for physical activity: acceptance				
T0	745	1.22 (0.74)	789	1.21 (0.71)
T1	676	1.11 (0.76)	695	1.09 (0.73)

**Fig. 3:**
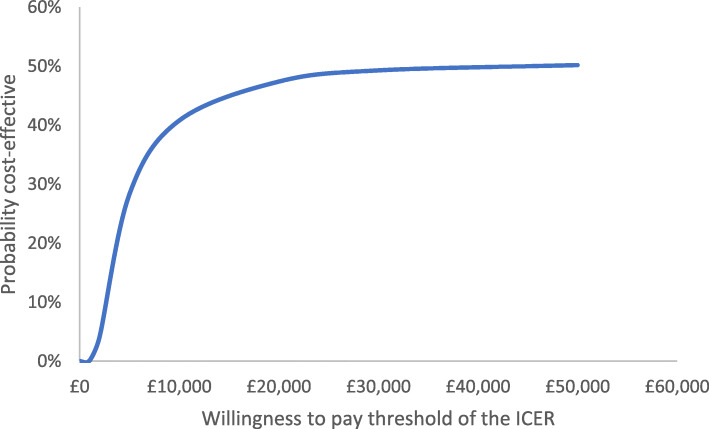
Cost-effectiveness acceptability curve

The mean cost of the PLAN-A intervention was £2817 per school, equivalent to £31 per Year 9 girl (Table 5). In unadjusted analyses, there was some evidence that KIDSCREEN-10 and EQ-5D-Y VAS scores were better in the intervention group (Table 6). However, differences were small and consistent with no effect of the intervention in adjusted analyses. The cost-effectiveness acceptability curve (Fig. 3) shows that, had the intervention been effective at increasing the primary outcome, there is a relatively low (47 %) probability that the intervention is cost-effective when the willingness to pay by the decision maker is £20,000 per QALY. The cost per unit change in weekday MVPA was negative £-37.34 (95 % CI: -£94.30 to £72.15) reflecting the lack of effectiveness. There is also a very low probability of being cost-effective over a range of cost per MVPA willingness to pay thresholds (web appendix 4).

**Table 5:** Intervention set up and delivery costs

Type of cost^a^	Cost per school	Cost per girl ^b^
Total cost per Train the trainer event	£319.09	£3.53
Total cost intervention consumables^c^	£250.76	£2.77
Peer nomination	£163.55	£1.81
Peer supporter training with pupils (two-day training and single top-up day)	£1393.56	£15.42
Co-ordination of intervention delivery	£93.67	£1.04
School staff time (arranging/ attending training, peer nomination and intervention delivery)	£595.88	£6.59
**Total**^**d**^	£2816.51	£31.16

^a^ all are Local authority costs except School staff costs

^b^ Per girl in year 9 on register at T0 (904 girls on roll in intervention schools at T0, including 146 who did not consent to take part in trial data collection)

^c^ Includes all materials and printing resource for the Plan-A intervention

^**d**^ Numbers may differ from summed totals due to rounding

**Table 6:** Quality of Life scores, QALYs and costs

	Control	Intervention	Differences between groups (95 % CI) (= Incremental costs/ effects)	
	Mean (sd)	Mean (sd)	Unadjusted (1000 bootstrapped 95 % CI)	Adjusted for baseline level and stratification variables^a^
KIDSCREEN-10 change score T0 to T1 (*n* = 694, 680) ^b^	-0.340 (0.830)	-0.229 (0.888)	0.111 (0.016 0.195)	0.095 (-0.120, 0.202)
CHU-9D change score T0 to T1 (*n* = 676,662) ^b^	-0.030 (0.077)	-0.025 (0.081)	0.005 (-0.004, 0.014)	0.004 (-0.006, 0.015)
EQ-5D-Y VAS change score T0 to T1 (*n* = 678, 656) ^b^	-4.643 (22.999)	-2.228 (21.678)	2.405 (0.106, 4.705)	1.136 (-2.807, 5.080)
QALYs (*n* = 676, 662) ^b^	0.822 (0.088)	0.822 (0.087)	0.001 (-0.009, 0.010)	0.002 (-0.003, 0.007)
Per-pupil intervention cost (*n* = 937, *n* = 904) ^b^	nil	£31.16	£30.50	N/A

Note: Patient reported outcome, utility and QALY measures are reported to 3 decimal places due to the small differences

^a^ adjusted for baseline level and the stratification variables (area and weighted IMD)

^b^ All are complete cases (pupils with measurements at T0 and T1) with the exception of per-pupil costs, which related to the total number of girls on the roll at the start of year 9

## Discussion

The data presented in this paper provide clear evidence that the PLAN-A intervention program did not result in increased levels of weekday or weekend MVPA or reductions in sedentary time among adolescent girls. This result also means that PLAN-A did not lead to demonstrable cost-effectiveness in terms of cost per unit change in QALY. Further analyses showed that there was evidence of differential treatment effects by proportion of sedentary time at baseline which could suggest that the PLAN-A intervention was more effective in pupils who were highly inactive, however the analysis was not powered to detect differences in this subgroup analysis so estimates and confidence intervals should be interpreted with caution. The mixed-methods process evaluation (which will be published in full elsewhere) showed that the intervention was delivered with a high degree of implementation and theoretical fidelity and enjoyed by the attendees, demonstrating that peer-led interventions such as PLAN-A can be delivered with good fidelity and at scale. However, the overall message of this study is that the PLAN-A intervention was not effective in improving weekday MVPA and there is no evidence to suggest it should be disseminated as a public health strategy to increase physical activity among adolescent girls.

The findings reported in this trial were surprising as the feasibility study, which was conducted in six secondary schools (4 intervention and 2 control), showed that the program yielded a difference of 6 min in weekday MVPA at the follow-up period favouring the intervention arm [10]. The relatively small size of the feasibility study necessitated a larger evaluation to be sure that the positive effects shown in the feasibility study were not simply down to chance and warranted widespread dissemination. There are several notable differences between the feasibility and definitive evaluation that may partially explain the divergent findings. First, the participant age group was changed from Year 8 to Year 9 to allow participants to provide their own consent and meet new ethical requirements that came into force as a result of the EU General Data Protection Regulation (GDPR) introduced in 2018. Changing the consent process affected the percentage of the eligible sample that participated in the study measures, dropping from 95 % in the feasibility study (range across schools = 89–98 %) to 78 % in the definitive trial (range across schools = 46–97 %). The possible implications of having less reach within the year group for measurements include (a) not measuring the effect of the intervention across a whole year group, and (b) there may have been peer-supporters trained whose peer groups did not provide data. Furthermore, school contacts expressed in post-intervention interviews that the intervention would be well suited to a younger year group, many of the Year 9 girls felt the content was repetitive and not sufficiently challenging, and two trainers who also delivered the intervention in the feasibility study perceived notably poorer engagement and attitudes in the older girls which affected delivery in some schools. Second, the schools that took part in this study were larger than in the feasibility study (mean n = 92 eligible girls per school in this trial vs. mean n = 75 in the feasibility trial) with comparable differences in the average number of girls in the year group who were trained as peer supporters (mean 16.40 vs. 13.75 respectively). This likely reduced the dose of training that each peer supporter received, especially in two schools where the number of peer supporters attending training exceeded 20. Third, two schools had all three of their intervention sessions delivered on the school site due to school-based logistical issues. This contravened the intervention model where off-site delivery of the training was designed (as in ASSIST) to provide a learning environment that felt distinct from school to remove distractions and enhance engagement. This was highlighted as important in the feasibility study, and process evaluation data indicate that the two schools that delivered training on-site experienced challenges to high-quality delivery and pupil engagement.

The findings reported in this paper are consistent with several recent papers that have reported that, with a few notable exceptions [34, 35], school-based physical activity interventions have had very limited impacts on the physical activity levels of adolescents and adolescent girls in particular. For example, Harrington [36] and colleagues reported that the Girls Active secondary school intervention, which provided support to schools to change their physical activity policies and culture did not have an impact on accelerometer-measured MVPA when assessed via a cluster randomised controlled trial in 20 schools at 14 month follow-up. Similarly, Corder [37] and colleagues reported that a physical activity program for Year 9 girls which focussed on older adolescent mentors and in-class peer leaders encouraging Year 9 girls to engage in 2 new weekly forms of physical activity yielded no impact on accelerometer measured MVPA at 10-month follow-up. Collectively, findings from these two UK-based definitive RCTs and our own, which all employed rigorous designs suggest that the traditional school-based physical activity intervention approaches designed to date are not effective or cost-effective at increasing physical activity, and that alternative approaches that look at different interventions and evaluation designs may be necessary [38].

Our findings are consistent with a recent meta-analysis by Beets [39] and colleagues which showed that nine different types of generalisability biases impacted on the success of scaling up obesity-related interventions from feasibility study to definitive trial. Two of these biases were likely to have been important in the scale up of PLAN-A; that there were changes in intervention support as the number of girls being trained changed, and there was a target audience bias as we had to change the year group from Year 8 to Year 9. This study also highlights that changes to external contexts (e.g. GDPR consent requirements) can also strongly influence the implementation of well-planned interventions. Although the change in age group was only a single school year, it is a time when girls are experiencing considerable change in friendship structures, prioritisation of competing activities, and level of autonomy over their physical activity and, crucially, it is not the year group with which the intervention was designed and piloted.

There was no evidence that the intervention was associated with changes in psychosocial mediators, neither did the mediators show any meaningful correlations with weekday MVPA. It is therefore possible that the intervention was not sufficient to impact on the hypothesised mediators and that as such the intervention did not function as per the hypothesized logic model. These findings are similar to the feasibility study [16] and will be expanded upon using qualitative process evaluation data in a future publication. Other studies have reported positive effects of SDT-based physical activity interventions on physical activity levels and mediators such as intrinsic motivation [40, 41], however the interventions in those studies provided short term and more direct or enhanced physical activity sessions (e.g., enhanced PE, walking interventions) delivered by adults, compared to the peer-led informal diffusion approach in PLAN-A. Further, these studies have various methodological shortcomings (e.g., small samples, no published protocol, non-randomised, self-reported physical activity) making comparisons difficult. Forthcoming systematic reviews of the evidence for the effects of school-based interventions on motivation for physical activity will shed more light on this area [42].

There was some evidence of an age-related decline in most of the psychosocial factors, which is consistent with the feasibility trial findings [16] and may indicate a general decrease in high quality motivation and physical activity related self-perceptions. These could be broadly reflective of the age-related decline in physical activity amongst girls [4–6].

### Strengths and limitations

The key strengths of this study are a function of the robust design. Physical activity was measured using accelerometery, schools were the unit of allocation and the intervention was delivered to an entire school group as opposed to a sub-group of participants. It is also important to reiterate that the intervention was co-developed with adolescent girls and built on a successful smoking cessation intervention and had shown considerable promise in a robustly-conducted feasibility study [10]. Although schools were sampled to reflect a broad range of socioeconomic positions this study was limited by the geographical focus which just included schools in southwest England. The study only included two measurement points and it is possible that an assessment closer to the end of the intervention period would have provided evidence of an immediate intervention impact. It is also important to clarify that although our analysis highlighted that we still had ample power to detect an intervention effect complete accelerometer data were only collected on 78 % of participants at both T0 and T1. While we think that these missing data are unlikely to have altered the interpretation of the study findings it is important to recognize that these data were missing.

## Conclusions

This study has shown that the PLAN-A intervention did not result in higher levels of weekday MVPA or associated secondary outcomes among Year 9. The PLAN-A intervention should not be disseminated as a public health strategy and alternative approaches to increasing physical activity among this group are needed.

## Supplementary information

**Additional file 1: **

## Data Availability

The datasets generated during the current study are not publicly available due as the project is ongoing and data are not ready for archiving. We plan on making the data available in the University of Bristol repository (https://data.bris.ac.uk/data/) once the final report has been published which we envisage will be by the end of 2021.

## Abbreviations

MVPA: Moderate-to-vigorous physical activity
ASSIST: A Stop Smoking In Schools Trial
PS: Peer supporter
DOI: Diffusion of Innovations
SDT: Self-Determination Theory
PLAN-A: Peer-Led physical Activity iNtervention for Adolescent girls
BRTC: Bristol Randomised Trials Collaboration
BREQ-2: Behavioural Regulation in Exercise Questionnaire-2
IMD: Index of Multiple Deprivation
ITT: Intention to Treat
ICER: Incremental Cost Effectiveness Ratio
CONSORT: Consolidated Standards of Reporting Trials
