# Supplementary material for: Effectiveness and cost-effectiveness of the PLAN-A intervention, a peer led physical activity program for adolescent girls: results of a cluster randomised controlled trial
Source: Int J Behav Nutr Phys Act. 2021 May 13;18:63. doi: 10.1186/s12966-021-01133-8 (PMC8117648; doi:10.1186/s12966-021-01133-8)
Supplement: Supplementary file 1 — Additional file 1: [file 12966_2021_1133_MOESM1_ESM.docx]

**Web appendix 1. Logic model for PLAN-A and hypothesised psychosocial mediators**

**OUTCOMES**

- Objectively-measured weekday/weekend physical activity
- Self-esteem
- Quality of life
- Attitudes towards physical activity
- Cost-effectiveness
- Lifestyle change

**OUTPUTS**

- N Trainers trained
- N PS recruited
- N PS completed training
- Selected Process measures (e.g., Fidelity, Evidence of peer support)
- Cost of intervention delivery

**ACTIVITIES**

- Peer nomination
- Peer Supporter (PS) briefing
- Train the trainers event (3 days)
- PS training 2 days plus top up

**INPUTS**

- Financial

(Sport England intervention costs funding)

- Human Resource

(to do PS nomination, Trainers, people who train the trainers, school key contacts)

- Time for prep and delivery of training & train the trainers
- Physical resources

(Access to schools for PS nomination and meetings, training venues)

- Logistics

(travel to venues, food etc)

- Resources

(manuals, kit for PS training, etc)

PS = Peer supporter

**Hypothesised psychosocial mediators**

| Physical activity motivation: Autonomous |
| --- |
| Physical activity motivation: Controlled |
| Physical activity psychological need satisfaction: Autonomy |
| Physical activity psychological need satisfaction: Competence |
| Physical activity psychological need satisfaction: Relatedness |
| PA self-efficacy |
| Physical activity social support |
| Peer norms for physical activity: prevalence |
| Peer norms for physical activity: importance |
| Peer norms for physical activity: acceptance |

**Web-Appendix 2– Imputation results and analysis of counts per minute in place of weekday MVPA**

Comparison of results of ITT analysis of complete cases with ITT analysis where missing data were imputed using multiple imputation for the primary outcome of MVPA on weekdays (mean)

|  | **n** | **Difference in means^a^** | **95% CI** | **p-value** |
| --- | --- | --- | --- | --- |
| Complete case | 1062 | -3.57 | (-6.75, -0.39) | 0.028 |
| Multiple imputation^b^ | 1558 | -2.54 | (-5.67, 0.59) | 0.110 |

*^a^ Adjusted for baseline MVPA on weekdays, number of days with valid accelerometer data, stratification variables and additional variables showing an imbalance between groups at baseline*

*^b^The imputation model included: baseline weekday MVPA, baseline sedentary time, family affluence, deprivation, method of travel to school, free school meals, indicators of school, area and arm. number of valid days of data, self-esteem, physical activity motivation, psychosocial need satisfaction, physical activity social support, peer norms for physical activity (prevalence and importance)and the KIDSCREEN outcome.* **Analysis of counts per minute (CPM)**

|  | **n** | **Mean** | **SD** |
| --- | --- | --- | --- |
| **Weekdays** | | | |
| Intervention | 603 | 354.55 | 119.24 |
| Control | 616 | 382.13 | 178.36 |
| Difference in means (95% CI)^a^: | -22.23 (-44.43, -0.05) | | p-value = 0.05 |
| Difference in means (95% CI)^b^: | -26.27 (-45.29, -7.25) | | p-value = 0.007 |
| **Weekends** | | | |
| Intervention | 347 | 376.29 | 364.58 |
| Control | 386 | 352.60 | 364.61 |
| Difference in means (95% CI)^a^: | -24.22 (-111.77, 63.34) | | p-value = 0.588 |
| Difference in means (95% CI)^b^: | -16.74 (-96.86, 63.38) | | p-value = 0.682 |
| **Average CPM across all valid days** | | | |
| Intervention | 603 | 357.06 | 126.57 |
| Control | 616 | 379.15 | 198.85 |
| Difference in means (95% CI)^a^: | -20.41 (-41.57, 0.75) | | p-value = 0.059 |
| Difference in means (95% CI)^b^: | -21.54 (-40.79, -2.29) | | p-value = 0.028 |

*^a^ ITT analysis adjusted for the outcome at baseline, the number of days of valid data and the stratification variables*

*^b^ ITT analysis additionally adjusted for additional variables that show an imbalance between groups at baseline*

**Web Appendix 3: Mediation analyses**

1. *Correlation between mediators and weekday MVPA over the study*

|  | **T0** | **T1** |
| --- | --- | --- |
| Physical activity motivation: Autonomous | 0.1228 | 0.0703 |
| Physical activity motivation: Controlled | -0.0003 | 0.0132 |
| Physical activity psychological need satisfaction: Autonomy | 0.0945 | 0.0185 |
| Physical activity psychological need satisfaction: Competence | 0.1046 | 0.0557 |
| Physical activity psychological need satisfaction: Relatedness | 0.0369 | 0.0338 |
| PA self-efficacy | 0.0844 | 0.0589 |
| Physical activity social support | 0.0529 | 0.041 |
| Peer norms for physical activity: prevalence | 0.0553 | 0.0597 |
| Peer norms for physical activity: importance | 0.0184 | 0.0242 |
| Peer norms for physical activity: acceptance | 0.0459 | 0.0073 |

*b) Mediated effects of the intervention on the primary outcome, by mediator*

| **Outcome at T1** | **Mediator** | **Average Direct Effect (95% CI)** | **Average Causal Mediated Effect (95% CI)** | **Total effect (95% CI)** | **Proportion of effect mediated, (95% CI)** |
| --- | --- | --- | --- | --- | --- |
| **Weekday MVPA** | Physical activity motivation:  Autonomous | -2.89  (-5.23, -0.47) | -0.02  (-0.18, 0.12) | -2.92  (-5.21, -0.56) | 0.01  (0.004, 0.04) |
|  | Physical activity motivation: Controlled | -2.62  (-4.90, -0.27) | -0.0002  (-0.16, 0.14) | -2.62  (-4.93, -0.23) | 0.00  (0.00, 0.00) |
|  | Physical activity psychological need satisfaction: Autonomy | -2.80  (-5.15, -0.36) | 0.02  (-0.08, 0.15) | -2.77  (-5.16 -0.29) | -0.01  (-0.04, -0.004) |
|  | Physical activity psychological need satisfaction:  Competence | -2.74  (-5.10, -0.30) | -0.02  (-0.16, 0.10) | -2.76  (-5.08, -0.37) | 0.01  (0.004, 0.04) |
|  | Physical activity psychological need satisfaction:  Relatedness | -2.80  (-5.21, -0.31) | -0.003  (-0.10, 0.10) | -2.80  (-5.20, -0.33) | 0.00  (0.00, 0.01) |
|  | PA self-efficacy | -2.78  (-5.16, -0.33) | -0.003  (-0.07, 0.06) | -2.79  (-5.15, -0.35) | 0.001  (0.001, 0.01) |
|  | Physical activity social support | -2.75  (-5.33, -0.08) | 0.02  (-0.11, 0.19) | -2.73  (-5.24, -0.12) | -0.01  (-0.06, -0.004) |
|  | Peer norms for physical activity: prevalence | -2.57  (-4.99, -0.06) | -0.003  (-0.12, 0.10) | -2.57  (-4.98, -0.08) | 0.001  (0.001, 0.01) |
|  | Peer norms for physical activity: importance | -2.75  (-5.12, -0.31) | -0.004  (-0.21, 0.21) | -2.76  (-5.04, -0.40) | 0.001  (0.001, 0.01) |
|  | Peer norms for physical activity: acceptance | -2.54  (-4.96, -0.04) | -0.02  (-0.17, 0.09) | -2.56  (-4.97, -0.08) | 0.01  (0.004, 0.06) |

*The average direct effect is the effect of the intervention on weekday MVPA which is unexplained by the mediators (the amount of the total effect which is not explained by the mediator). The average causal mediated effect is the effect of the intervention on the weekday MVPA which is explained by the mediator (the amount of the total effect which can be explained by the mediator). For instance, the first row of the table indicates that 1% of the intervention effect on MVPA is likely to be mediated by autonomous motivation*.

**Web Appendix 4: Cost effectiveness acceptability curve**

*Cost-effectiveness acceptability curve over a range of hypothetical willingness to pay thresholds for MVPA*
